# Identifying Parkinson's disease and parkinsonism cases using routinely collected healthcare data: A systematic review

**DOI:** 10.1371/journal.pone.0198736

**Published:** 2019-01-31

**Authors:** Zoe Harding, Tim Wilkinson, Anna Stevenson, Sophie Horrocks, Amanda Ly, Christian Schnier, David P. Breen, Kristiina Rannikmäe, Cathie L. M. Sudlow

**Affiliations:** 1 College of Medicine & Veterinary Medicine, University of Edinburgh, Edinburgh, United Kingdom; 2 Centre for Clinical Brain Sciences, University of Edinburgh, Edinburgh, United Kingdom; 3 Centre for Medical Informatics, Usher Institute of Population Health Sciences and Informatics, University of Edinburgh, Edinburgh, United Kingdom; 4 Institute of Genetics and Molecular Medicine, University of Edinburgh, Edinburgh, United Kingdom; 5 Centre for Cognitive Ageing and Cognitive Epidemiology, Edinburgh, United Kingdom; 6 Anne Rowling Regenerative Neurology Clinic, University of Edinburgh, Edinburgh, Scotland; Liverpool John Moores University, UNITED KINGDOM

## Abstract

**Background:**

Population-based, prospective studies can provide important insights into Parkinson’s disease (PD) and other parkinsonian disorders. Participant follow-up in such studies is often achieved through linkage to routinely collected healthcare datasets. We systematically reviewed the published literature on the accuracy of these datasets for this purpose.

**Methods:**

We searched four electronic databases for published studies that compared PD and parkinsonism cases identified using routinely collected data to a reference standard. We extracted study characteristics and two accuracy measures: positive predictive value (PPV) and/or sensitivity.

**Results:**

We identified 18 articles, resulting in 27 measures of PPV and 14 of sensitivity. For PD, PPV ranged from 56–90% in hospital datasets, 53–87% in prescription datasets, 81–90% in primary care datasets and was 67% in mortality datasets. Combining diagnostic and medication codes increased PPV. For parkinsonism, PPV ranged from 36–88% in hospital datasets, 40–74% in prescription datasets, and was 94% in mortality datasets. Sensitivity ranged from 15–73% in single datasets for PD and 43–63% in single datasets for parkinsonism.

**Conclusions:**

In many settings, routinely collected datasets generate good PPVs and reasonable sensitivities for identifying PD and parkinsonism cases. However, given the wide range of identified accuracy estimates, we recommend cohorts conduct their own context-specific validation studies if existing evidence is lacking. Further research is warranted to investigate primary care and medication datasets, and to develop algorithms that balance a high PPV with acceptable sensitivity.

## Introduction

Despite well-established pathological features, the aetiologies of Parkinson’s Disease (PD) and other parkinsonian conditions remain poorly understood and disease-modifying treatments have proved elusive[1]. Large, prospective, population-based cohort studies with biosample collections (e.g., UK Biobank, German National Cohort, US Precision Medicine Initiative) provide a robust methodological framework with statistical power to investigate the complex interplay between genetic, environmental and lifestyle factors in the aetiology and natural history of neurological disorders such as PD and other parkinsonian disorders[2–4].

Linkage to routinely collected healthcare data–which are administrative datasets collected primarily for healthcare purposes rather than to address specific research questions[5]–provides an efficient means of long term follow-up in order to identify large numbers of incident cases in such studies[2]. Furthermore, participant linkage to such datasets can be used in randomised controlled trials as a cost-effective and comprehensive method of follow-up for disease outcomes[6]. These data are coded using systems such as the International Classification of Diseases (ICD)[7], the Systematized Nomenclature of Medicine–Clinical Terms (SNOMED-CT) system[8], and the UK primary care Read system[9].

There are several mechanisms by which inaccuracies can arise when using routinely collected healthcare data to identify PD outcomes. False positives (participants who receive a disease code but do not have the disorder) may arise if a clinician incorrectly diagnoses the condition. Given that PD and other parkinsonian disorders are largely clinical diagnoses made without a definitive diagnostic test, there is the potential for diagnostic inaccuracies. Clinicopathological studies have shown discrepancies between clinical diagnoses in life and neuropathological confirmation[10] and there is evidence that accuracy increases when diagnoses are made by movement disorder specialists[11–13]. Secondly, diagnoses may be incorrectly recorded in medical records, or errors may arise during the coding process. Similarly, false negatives (patients who have the condition but do not receive a code) may arise due to under-diagnosis, omission of the diagnosis from the medical records (e.g., because the condition is not the primary reason for hospital admission), or errors during the coding process.

As a result, before such datasets can be used to identify PD and parkinsonism cases in prospective studies, their accuracy must be determined. Important measures are the positive predictive value (PPV, the proportion of those coded positive that are true disease cases) and sensitivity (the proportion of true disease cases that are coded positive). Specificity and negative predictive value are less relevant metrics in this setting. A high specificity (the proportion of those without the disease that do not receive a disease code) is important to ensure a high PPV, thereby minimising bias in effect estimates. With an appropriately precise choice of codes, the specificity of routinely collected healthcare data to identify disease cases in population-based studies is usually very high (98–100%)[14,15]. However, in a population-based cohort study where the overall prevalence of a disease is low, a high specificity does not guarantee a high PPV—a large absolute number of people without the disease can be incorrectly classified as being disease cases (false positives), yet the overall proportion of misclassified cases can be low (high specificity, low PPV)[16]. NPV, like PPV, is related to disease prevalence and will therefore be high in population-based studies where most individuals do not develop the disease of interest[14].

Previous systematic reviews on the accuracy of routine data to identify other neurological diseases such as stroke[14], dementia[17] and motor neurone disease[18] have summarised the existing literature and identified methods by which accuracy can be improved, as well as areas for further evaluation. Here, we systematically reviewed published studies that evaluated the accuracy of routinely collected healthcare data for identifying PD and parkinsonism cases.

## Methods

### Study reporting

We followed the Preferred Reporting Items for Systematic Review and Meta-analysis statement (PRISMA) guidelines for the reporting of this systematic review[19].

### Study protocol

We used the PRISMA Protocols (PRISMA-P) guideline to aid in the design of this study[20], and prospectively published the protocol (number: CRD42016033715, www.crd.york.ac.uk/PROSPERO/display_record.php?ID = CRD42016033715) [21].

### Search strategy

We (AS & TW) searched the electronic databases MEDLINE (Ovid), EMBASE (Ovid), CENTRAL (Cochrane Library) and Web of Science (Thomson Reuters) for relevant articles published in any language between 01.01.1990 and 23.06.2017. Our search strategy is outlined in S1 File. We chose the date limits based on our judgement that accuracy estimates from studies published prior to 1990 would have limited current applicability. We did not exclude studies based on the dates covered by the datasets. We also screened bibliographies of included studies and relevant review papers to identify additional publications.

### Eligibility criteria

To be included, studies had to have: compared codes for PD or parkinsonism from routinely collected healthcare data to a clinical expert-derived reference standard, and provide either a PPV and/or a sensitivity estimate (or sufficient raw data to calculate these). We excluded studies with <10 coded cases, due to the limited precision of studies below this size[17,18]. Studies reporting sensitivity values had to be population-based (i.e. community-based as opposed to hospital-based) with comprehensive attempts to detect all disease cases. Where multiple studies investigated overlapping populations, we included the study with the larger population size. Where articles assessed more than one dataset or evaluated both PPV and sensitivity, we included these as separate studies. Hereafter, we will refer to published papers as ‘articles’ and these separate analyses as ‘studies’.

### Study selection

Two authors (AS and SH) independently screened all titles and abstracts generated by the search, and reviewed full text articles of all potentially eligible studies to determine if the inclusion criteria were met. In the case of disagreement or uncertainty, we reached a consensus through discussion and, where necessary, involvement of a senior third author (CLMS).

### Data extraction

Using a standardized form, two authors (TW and ZH) independently extracted the following data from each study: first author; year of publication; time period during which coded data were collected; country of study; study population; average age of disease cases (or, if this was unavailable, the ages of participants at recruitment); study size (defined as the total number of code positive cases for PPV [true positives plus false positives] and the total number of true positives for sensitivity [true positives and false negatives]); type of routine data used (e.g., hospital admissions, mortality or primary care); coding system and version used; specific codes used to identify cases; diagnostic coding position (e.g. primary or secondary position); parkinsonian subtypes investigated; and the method used to make the reference standard diagnosis.

We recorded the reported PPV and/or sensitivity estimates, as well as any corresponding raw data. After discussion, any remaining queries were resolved with a senior third author (CLMS). When necessary, we contacted study authors to request additional information.

### Quality assessment

We adapted the Quality Assessment of Diagnostic Accuracy Studies 2 (QUADAS-2)[22] tool to evaluate the risk of bias in the estimates of accuracy and any concerns about the applicability of each article to our specific research question (S2 File). Two authors (TW and ZH) independently assigned quality ratings, with any discrepancies resolved through discussion. We performed this evaluation in the context of our specific review question and not as an indication of the overall quality of the articles. We assessed risk of bias at the article level rather than study level, as the methods for each study within an article were very similar. We did not exclude studies based on their quality assessment ratings, but rather considered a given study’s results in the context of the article’s risk of bias and applicability concerns. Where articles deemed to be at low of bias and articles at high risk of bias reported PPV or sensitivity estimates on the same type of dataset, we compared the reported estimates to assess the potential effect of bias on accuracy estimates.

### Statistical analysis/data synthesis

We tabulated the extracted data, and calculated 95% confidence intervals for the accuracy measures from the raw data using the Clopper-Pearson (exact) method. Due to substantial heterogeneity in study settings and methodologies, we did not perform a meta-analysis, as we considered any summary estimate to be potentially misleading. Instead, we assessed the full range of results in the context of study methodologies, populations and specific data sources. We also reported any within-study comparisons in which a single variable was changed to examine its effect on PPV or sensitivity. We performed analyses using the statistical software StatsDirect3.

## Results

### Study characteristics

**Fig 1:**
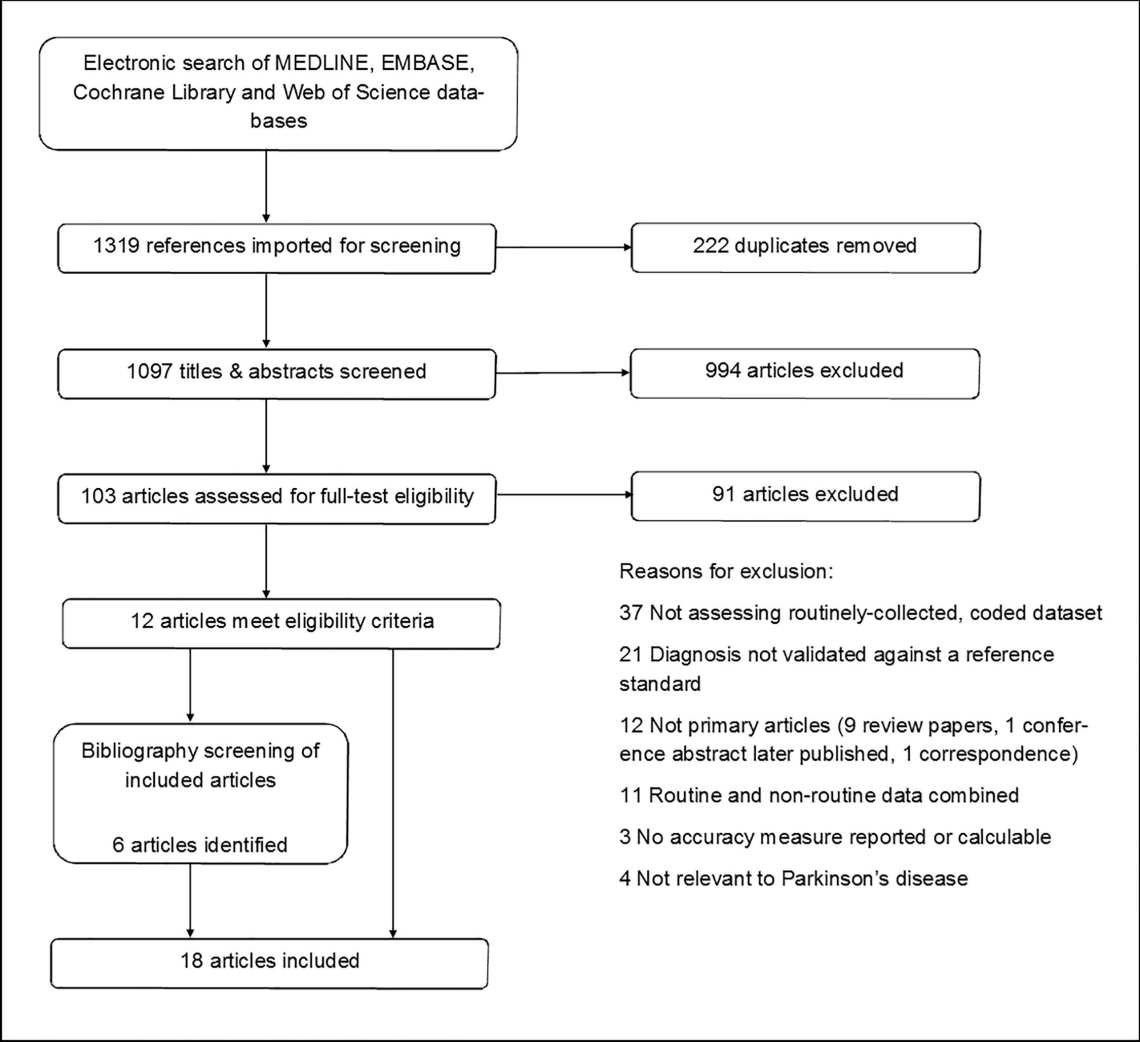
PRISMA flow diagram.

From an initial 1319 identified articles, we removed 222 duplicates and excluded 994 considered to be irrelevant after screening the titles and abstracts. We therefore examined the full text articles for 103 papers. Of these, we excluded 37 that did not assess the accuracy of a routinely collected, coded dataset, 21 that did not validate the coded data against any reference standard, 12 that were not primary research studies, 11 that combined routine and non-routine data, three where no accuracy measure was reported or calculable, and four that did not assess coding in PD. 18 published articles fulfilled our inclusion criteria[23–40]. A flow diagram of the study selection process is shown in Fig 1. We obtained key additional information from the authors of two studies[32,36]. Of the 18 included articles, 13 reported PPV[23,25–36], four reported sensitivity[37–40] and one reported both[24]. Four articles contained more than one study[23–25,29]. One of these consisted of multiple sub-studies, using different methods to evaluate datasets across several countries, so we included these as six separate studies[25]. In total, there were 27 measures of PPV and 14 of sensitivity. Study characteristics are summarised in Tables 1 and 2 respectively.

**Table 1:** Characteristics of studies reporting positive predictive value, stratified by dataset type.

First author & year of publication	Year of study	Country	Study population composition	Age*	Proportion male	Study size (n)	Routine dataset used	Coding system	Codes used to identify cases	Diagnostic coding position	Reference standard
**Hospital-derived datasets:**											
Butt 2014^[^^23^^]^	1991–2011	Canada	Population of Ontario ≥20yrs	Mean 74	60	Inpatient: 79 Outpatient: 435	Hospital: inpatient Hospital: outpatient	ICD-9 (pre-2002) ICD-10 (post-2002)	Parkinsonism: ICD-9: 332.0, 332; ICD-10: G20, G21.0–0.4, G21.8–9, G22, F02.3	Not specified	Medical record review
Feldman 2012^[^^24^^]^	1964–2004	Sweden	Twins across Sweden >50yrs	Mean 75	Unclear	PD: 72 Parkinsonism: 75	Hospital: inpatient	ICD-7 (1961–67) ICD-8 (1968–86) ICD-9 (1987–96) ICD-10 (1997–2009)	PD: ICD-7: 350; ICD-8: 342.00; ICD-9: 332.0; ICD-10: G20 Parkinsonism: ICD-8: 342.08, 342.09; ICD-9: 333.0; ICD-10: G21.4, G21.8, G21.9, G23.1, G23.2, G23.9, G25.9	Any	Screening interview, medical record review and examination by physician
Gallo [a] 2015^[^^25^^]^	1994–2010	Sweden	Hospital patients, EPIC study participants	Mean 46 at recruitment	Unclear	62	Hospital: unclear	ICD-9 (pre-1996) ICD-10 (post-1996)	PD: ICD-9: 332; ICD-10: G20, G21	Not specified	Medical record review
Gallo [b] 2015^[^^25^^]^	1991–2010	Sweden	Hospital patients, EPIC study participants	Mean 58 at recruitment	Unclear	299	Hospital: inpatient and outpatient	ICD-9 ICD-10	PD: ICD-9: 332; ICD-10: G20	Not specified	Medical record review
Kestenbaum 2015^[^^26^^]^	2009–2014	USA	Tertiary referral centre patients	Unclear	Unclear	100	Hospital: unclear	ICD-9	PD: 332.0	Not specified	Medical record review
Swarztrauber 2005^[^^27^^]^	1998–2002	USA	Veterans hospital patients	Mean 76	98	175	Hospital: inpatient and outpatient	ICD-9-CM	Parkinsonism: 332.0, 332.1, 333.0	Not specified	Medical records review
Szumski 2009^[^^28^^]^	2001–2004	USA	Veterans hospital patients	Mean 76	98	577	Hospital: outpatient	ICD-9-CM	PD: 332.0	Not specified	Medical record review
Wei 2016^[^^29^^]^	Unclear	USA	Hospital patients	Unclear	Unclear	100	Hospital: inpatient and outpatient	ICD-9	PD: 332.0	Not specified	Medical record review
Wermuth 2015^[^^30^^]^	1996–2009	Denmark	Neurological hospital patients	Median 55–64	59	2625	Hospital: inpatient and outpatient	ICD-8 ICD-10	PD: ICD-8: 342, ICD-10: G20	Primary	Medical record review
White 2007^[^^31^^]^	1998–2000	USA	Veterans hospital patients	Median 75	96	782	Hospital: inpatient and outpatient	ICD-9-CM	Parkinsonism: 332.0, 332.1	Any	Medical record review
**Primary care-derived datasets:**											
Hernán 2004^[^^32^^]^	1995–2001	UK	GP patients	Unclear	Unclear	106^†^	Primary care	Read code	Not specified (investigated PD)	Not applicable	Medical record review
**Prescription-derived datasets:**											
Butt 2014^[^^23^^]^	1991–2011	Canada	Population of Ontario ≥65 years	Mean 74	60	395	Prescriptions	Not specified	Parkinsonism: Levodopa; MAO-B inhibitors; dopamine agonists; COMT inhibitors	Not applicable	Medical record review
Meara 1999^[^^33^^]^	Not stated	UK	GP patients	Mean 76	Unclear	PD: 402 Parkinsonism: 402	Prescriptions (from primary care)	Not specified	PD: Not specified Parkinsonism: Not specified	Not applicable	History and examination by physician and medical record review
Wei 2016^[^^29^^]^	Unclear	USA	Hospital patients	Unclear	Unclear	100	Prescriptions	Not specified	PD: Rotigotine; Entacapone; Selegiline hydrochloride; Pergolide; Rasagiline	Not applicable	Medical record review
**Mortality datasets:**											
Feldman. A 2012^[^^24^^]^	1998–2007	Sweden	Twins across Sweden >50yrs	Mean 75	Unclear	PD: 18 Parkinsonism: 18	Mortality	ICD-10	PD: G20 Parkinsonism: G21.4, G21.8, G21.9, G23.1, G23.2, G23.9, G25.9	Any	Screening interview, medical record review and examination by physician
**Combined datasets (accuracy measures for constituent datasets unable to be separated):**											
Bower 1999^[^^34^^]^	1976–1990	USA	Population of Olmsted county	Unclear	Unclear	2472	Synthesised medical information	H-ICDA	Parkinsonism: H-ICDA 53 diagnostic codes	Not specified	Medical record review
Gallo [c] 2015^[^^25^^]^	1998–2010	Spain	EPIC study participants	Mean 50 at recruitment	49	39	Prescriptions; Primary care; Mortality; Hospital: inpatient	ATC/DDD index ICD-9	PD: ICD-9: 332, 332.0, 332.1; ATC/DDD index N04, N04A, N04B	Not specified	Medical record review
Gallo [d] 2015^[^^25^^]^	Unclear—2010	Spain	EPIC study participants	Mean 50 at recruitment	48	41	Primary care; Prescriptions; Mortality	ICPC ATC/DDD index ICD-9	PD: ICPC N87; ATC/DDD index N04, N04A, N04B; ICD-9: 332.x	Not specified	Medical record review
Gallo [e] 2015^[^^25^^]^	1998–2010	Spain	EPIC study participants	Mean 49 at recruitment	32	99	Hospital: inpatient; Primary care; Prescriptions; Mortality	ICD-9 ICPC2 ATC/DDD index ICD-10	PD: ICD-9: 332; ICPC2-WICC N87; ATC/DDD index N04x; ICD-10: G20	Not specified	Medical record review
Gallo [f] 2015^[^^25^^]^	1992–2008	Italy	EPIC study participants	Mean 50 at recruitment	57	81	Hospital: inpatient; Mortality; Prescriptions	ICD-9 ICD-10 ATC/DDD index	PD: ICD-9 332; ATC/DDD index: N04, N04A, N04B; ICD-10 G20	Not specified	Medical record review
Savica 2013^[^^35^^]^	1991–2005	USA	Population of Olmsted county	Unclear	Unclear	4957	Synthesised medical information	H-ICDA ICD-9	Parkinsonism: H-ICDA 38 diagnostic codes, ICD-9: 331.9, 332.0, 332.1, 333.0, 333.1, 781.0, 781.3	Not specified	Medical record review
Thacker 2016^[^^36^^]^	2005–2015	USA	Patients from a single medical institution	Unclear	Unclear	129	Hospital: inpatient and outpatient Primary care	ICD-9	PD: 332, 332.0	Primary	Medical record review

Year of study: the time period during which coded data was collected.

*—any information given regarding the ages of cases or age at recruitment. Study size: the total number of code positive cases (true positives plus false positives). Where both PD and parkinsonism were investigated in one article, study sizes for both are displayed. Study population composition: population cohort from which cases were identified.

ICD codes for Parkinson’s disease—ICD-7 350; ICD-8 342.00; ICD-9(-CM) 332.0; ICD-10 G20.

ICD codes for other Parkinsonism—ICD-8: 342.08 (other defined Parkinsonism), 342.09 (unspecified Parkinsonism); ICD-9(-CM): ICD-9-CM: 332.1 (secondary Parkinson’s disease), 333.0 (other degenerative diseases of the basal ganglia); ICD-10: G21.4 (vascular Parkinsonism), G21.8 (other defined secondary Parkinsonism), G21.9 (unspecified secondary Parkinsonism), G23.1 (progressive supranuclear ophthalmoplegia), G23.2 (striatonigral degeneration), G23.9 (unspecified degenerative disease of basal ganglia), G25.9 (unspecified extrapyramidal and movement disorder). Additional ICD codes–ICD-9: 331.9 (cerebral degeneration), 333.1 (essential and other specified forms of tremor), 781.0 (abnormal involuntary movements), 781.3 (lack of coordination).

^†^Exact study size unknown, reported as 7% of 1521 (could range from 99–115)–authors contacted, but data unavailable.

Abbreviations: PD—Parkinson’s Disease; EPIC—European Prospective Investigation into Cancer and Nutrition study; ICD- International Classification of Diseases; H-ICDA—Hospital Adaptation of ICDA; ATC/DDD index—Anatomical Therapeutic Chemical Classification System with Defined Daily Doses; ICPC—International Classification of Primary Care.

**Table 2:** Characteristics of studies reporting sensitivity, stratified by dataset type.

First author, year of publication	Year of study	Country	Study population composition	Age (years)*	Proportion male	Study size (n)	Routine dataset used	Coding system	Codes used to identify cases	Diagnostic coding position	Reference standard
**Mortality certificate-derived datasets:**											
Benito-Leόn 2014^[^^37^^]^	1994–2007	Spain	Three communities near Madrid	Mean 77	56%	82	Mortality	ICD-9 (pre 1999) ICD-10 (post 1999)	Not specified (investigated PD)	Primary	Screening (in-person, telephone and mail questionnaire) and neurological examination
Beyer 2001^[^^38^^]^	1993–1996	Norway	County (Rogaland)	Mean 79	Unclear	84	Mortality	ICD-9 or ICPC	Not specified (investigated PD)	Primary + Any	Semi-structured interview and a clinical examination
Fall 2003^[^^39^^]^	1989–1998	Sweden	Central district of Ӧstergӧtland	Mean 82	Unclear	121	Mortality	ICD-9	Not specified (investigated PD)	Primary + Any	Examination and medical record review
Feldman 2012^[^^24^^]^	1998–2008	Sweden	Twins across Sweden >50yrs	Mean 75	Unclear	PD: 77 Parkinsonism: 127	Mortality	ICD-10	PD: G20 Parkinsonism: G21.4, G21.8, G21.9, G23.1, G23.2, G23.9, G25.9	Any	Screening interview, medical record review and examination
Williams-Gray 2013^[^^40^^]^	2000–2012	UK	County (Cambridgeshire)	Mean 70	Unclear	63	Mortality	Not specified	Not specified (investigated PD)	Primary + Any	History and neurological examination
**Hospital-derived datasets:**											
Feldman 2012^[^^24^^]^	1964–2009	Sweden	Twins across Sweden >50yrs	Mean 75	Unclear	PD: 132 Parkinsonism: 194	Hospital: inpatient	ICD-7 (1961–67) ICD-8 (1968–86) ICD-9 (1987–96) ICD-10 (1997–2009)	PD: ICD-7: 350; ICD-8: 342.00; ICD-9: 332.0; ICD-10: G20 Parkinsonism: ICD-8: 342.08, 342.09; ICD-9: 333.0; ICD-10: G21.4, G21.8, G21.9, G23.1, G23.2, G23.9, G25.9	Any	Screening interview, medical record review and examination

Year of study: the time period during which coded data was collected.

*—any information given regarding the ages of cases or age at recruitment Study size: the total number of true positive according to the reference standard (true positives and false negatives). Where both PD and parkinsonism were investigated in one article, study sizes for both are displayed. Study population composition: population cohort from which cases were identified.

ICD codes for Parkinson’s disease—ICD-7 350; ICD-8 342.00; ICD-9 332.0; ICD-10 G20.

ICD codes for other Parkinsonism—ICD-8: 342.08 (other defined Parkinsonism), 342.09 (unspecified Parkinsonism); ICD-9: 333.0 (other degenerative diseases of the basal ganglia); ICD-10: G21.4 (vascular Parkinsonism), G21.8 (other defined secondary Parkinsonism), G21.9 (unspecified secondary Parkinsonism), G23.1 (progressive supranuclear ophthalmoplegia), G23.2 (striatonigral degeneration), G23.9 (unspecified degenerative disease of basal ganglia), G25.9 (unspecified extrapyramidal and movement disorder).

Study size varied considerably, ranging from 39–4957. All 18 articles were based in high-income countries. Three were from the UK[32,33,40], six from mainland Europe[24,25,30,37–39], eight from the USA[26–29,31,34–36], and one from Canada[23]. There were 12 PPV estimates and two sensitivity estimates from hospital data[23–31], two PPV and 10 sensitivity estimates from mortality data[24,37–40], two PPV estimates from primary care data[32], four PPV estimates from prescription data[23,29,33] and seven PPV estimates and two sensitivity estimates from combining datasets from different sources[24,25,34–36]. There were no sensitivity estimates from primary care or prescription data.

PD was evaluated in 13 articles, with eight estimating PPV[25,26,28–30,32,33,36], four estimating sensitivity[37–40] and one estimating both[24]. Parkinsonism was evaluated by seven articles, of which six estimated PPV[23,27,31,33–35] and one assessed both PPV and sensitivity[24]. All of the parkinsonism articles combined PD with other causes of parkinsonism.

The methods of reference standard used could be broadly divided into two categories: patient history and examination (5/5 articles reporting sensitivity) and medical record review (14/14 of articles reporting PPV). Three articles used in-person examination and medical record review in combination[24,33,39]. In addition, where entire populations were under study, some studies incorporated a screening method (e.g., telephone interview) to identify potential cases[24,37].

Where reported, codes used to identify PD cases were consistent and appropriate to the ICD version used. However, the range of codes used to identify other parkinsonian conditions varied considerably, reflecting the broad range of pathologies that can lead to parkinsonism. Seven studies did not specify the exact codes used[29,32,33,37–40]. ICD versions used reflected the time period over which the studies were conducted. 19 studies used ICD-9 (or ICD-9-CM, a clinically modified version used in the USA, and identical to ICD-9 with respect to parkinsonian diagnoses)[23–29,31,35–39], 11 used ICD-10[23–25,30,37], three used ICD-8[24,30], and two used ICD-7[24]. One of the primary care studies used Read-coded data[32]. Four studies, including the three that evaluated prescription data, did not specify the coding system used[23,29,33,40].

The diagnostic coding position assessed also varied. Three studies assessed primary diagnoses alone[30,36,37], eight used any diagnostic position[24,31,38–40], while 13 did not specify the coding position[23,25–29,34,35]. Diagnostic position was not applicable in the studies of primary care and prescription data due to the nature of these datasets[23,29,32,33].

### Quality assessment

Only two articles were judged to be of low risk of bias or applicability concerns in the QUADAS-2 assessment[23,24] (S1 Table). Across the risk of bias domains, the most common area of concern was inappropriate or unclear code lists to identify disease cases (10/18), followed by: selection bias (8/18), patient flow (i.e. inappropriate inclusions and exclusions or patients being lost to follow-up) (5/18) and insufficiently rigorous or unclear reference standards (4/18).

### Positive predictive value

**Fig 2:**
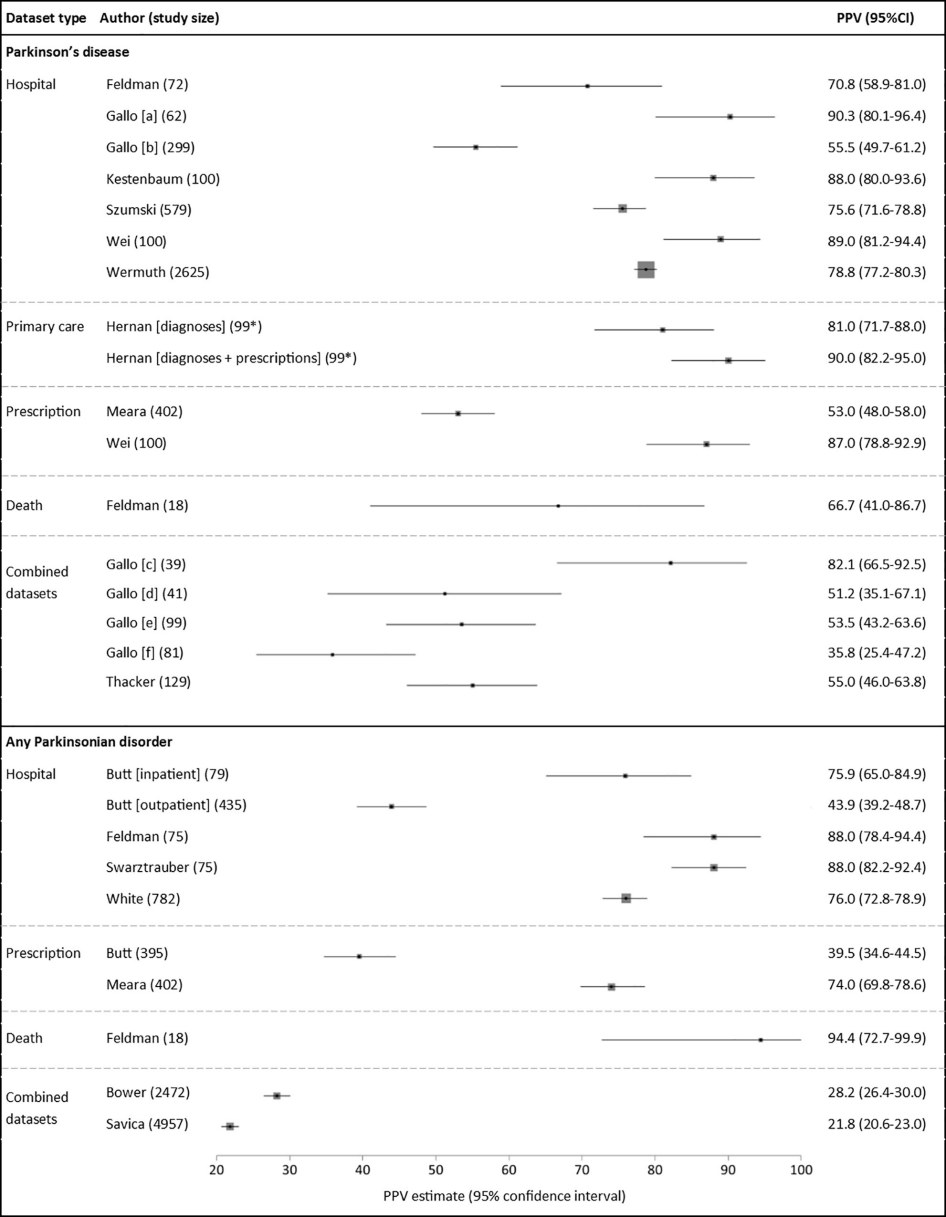
Positive predictive values (PPVs) of coded diagnoses. Study size: total number of code-positive cases (true positives + false positives). *Exact sample size unknown, most conservative estimate used. Box sizes reflect Mantel-Haenszel weight of study (inverse variance, fixed effects).

For PD, there were 17 PPV estimates in total (Fig 2)[24–26,28–30,32,33,36]. These comprised seven PPV estimates of hospital data alone[24–26,28–30], one of mortality data alone[24], two for prescription data alone[29,33], one of primary care data alone[32], one of prescription data and primary care data in combination[32], and five of datasets used in combination[25,36]. PPVs ranged from 36–90% across all studies. Nine of the 17 estimates were >75%. The single study of Read coding in primary care data alone reported a PPV of 81%, increasing to 90% with the presence of a relevant medication code in addition to a diagnostic code[32]. The two studies of medication data alone reported PPVs of 53% and 87%[29,33]. The single, small study of mortality data had a PPV of 67%[24].

One of the two articles judged to be at low risk of bias investigated the PPV of hospital admissions data to identify PD, reporting a PPV of 70.8%[24]. This value fell in between the range of other studies (range 55.5–90.3%), raising the possibility that estimates from studies at the extremes of the range may be influenced by bias.

Several within-study comparisons were available from three studies identifying PD (Table 3)[24,28,29]. Two of these investigated the change in PPV for hospital data to identify PD when algorithms containing additional criteria were used[24,28]. Both showed a moderate increase in PPV if a relevant diagnosis code was recorded more than once, or if a specialist department assigned such a code. One study reported an increase in PPV when only primary position diagnoses were assessed[24]. Another showed that incorporating selected medication codes with diagnosis codes increased the PPV from 76% to 86%, although this was at the expense of reduced case ascertainment[28]. Finally, one study showed that the combination of a diagnostic code in hospital data with a relevant medication code increased the PPV when compared to using either dataset alone (94% versus 87% and 89% respectively)[29].

**Table 3:** Within-study analyses. Algorithm development.

Criteria applied:	PPV % (95% CI)		Number of cases identified
**Parkinson’s Disease**			
a) **Feldman 2012** (hospital inpatient data)			
Parkinson’s disease ICD code only		71 (59–81)	72
Exclusion of patients with other (non-Parkinson’s disease) parkinsonian codes		70 (58–81)	67
Code frequency ≥2 hospital admissions		76 (61–88)	42
Code in primary diagnostic position		83 (70–92)	53
Code assigned in specialist department (neurological/neurosurgical/geriatric)		83(63–95)	24
b) **Szumski 2009** (hospital outpatient data)			
Parkinson’s disease ICD codes only		76 (72–79)	579
Code frequency ≥2 at any clinic		79(76–83)	409
Code assigned in any neurology clinic		79 (75–83)	352
Code assigned in movement disorder speciality clinic		87 (81–92)	177
Code + prescribed antiparkinsonian medication		86 (82–89)	408
c) **Wei 2016**			
Parkinson’s disease ICD codes only		89 (81–94)	100
Prescription only		87 (78–93)	100
ICD code and prescription		94*	Unknown*
**Parkinsonism**			
d) **Butt 2014**			
Hospital inpatient ICD code ever		87 (79–96)	63
Hospital outpatient ICD code ever		55 (49–60)	297
Prescription ever		40 (35–44)	395
Outpatient code frequency ≥2 in one year		83 (77–89)	169
Outpatient code frequency ≥2 in one year by a specialist		87 (81–92)	134
Outpatient code AND Prescription		85 (79–90)	174
Prescription AND outpatient code within +/- 6 months		87 (82–92)	166

The effect of additional criteria to identify PD cases on PPV and the number of cases identified.

* Sample size and confidence intervals unknown for this accuracy measure.

For parkinsonism, there were 10 PPV estimates in total (Fig 2)[23,24,27,31,33–35]. These comprised five estimates from hospital data alone[23,24,27,31], two from prescription data alone[23,33], one from mortality data alone[24], and two from using datasets in combination[34,35]. PPVs ranged from 40–94% in the single datasets and from 22–28% in the combination datasets. The two studies of parkinsonism in prescription data produced very different PPV estimates of 40% and 74%[23,33]. One of these studies reported that the PPV of medication data to identify any parkinsonian disorder was considerably higher than that for PD (74% and 53% respectively)[33].

The two articles with low risk of bias investigated the use of hospital admissions data to identify parkinsonism cases. These articles reported PPVs of 76%[23] and 88%[24], which is consistent with the values reported by other studies judged to be at risk of bias.

### Sensitivity

**Fig 3:**
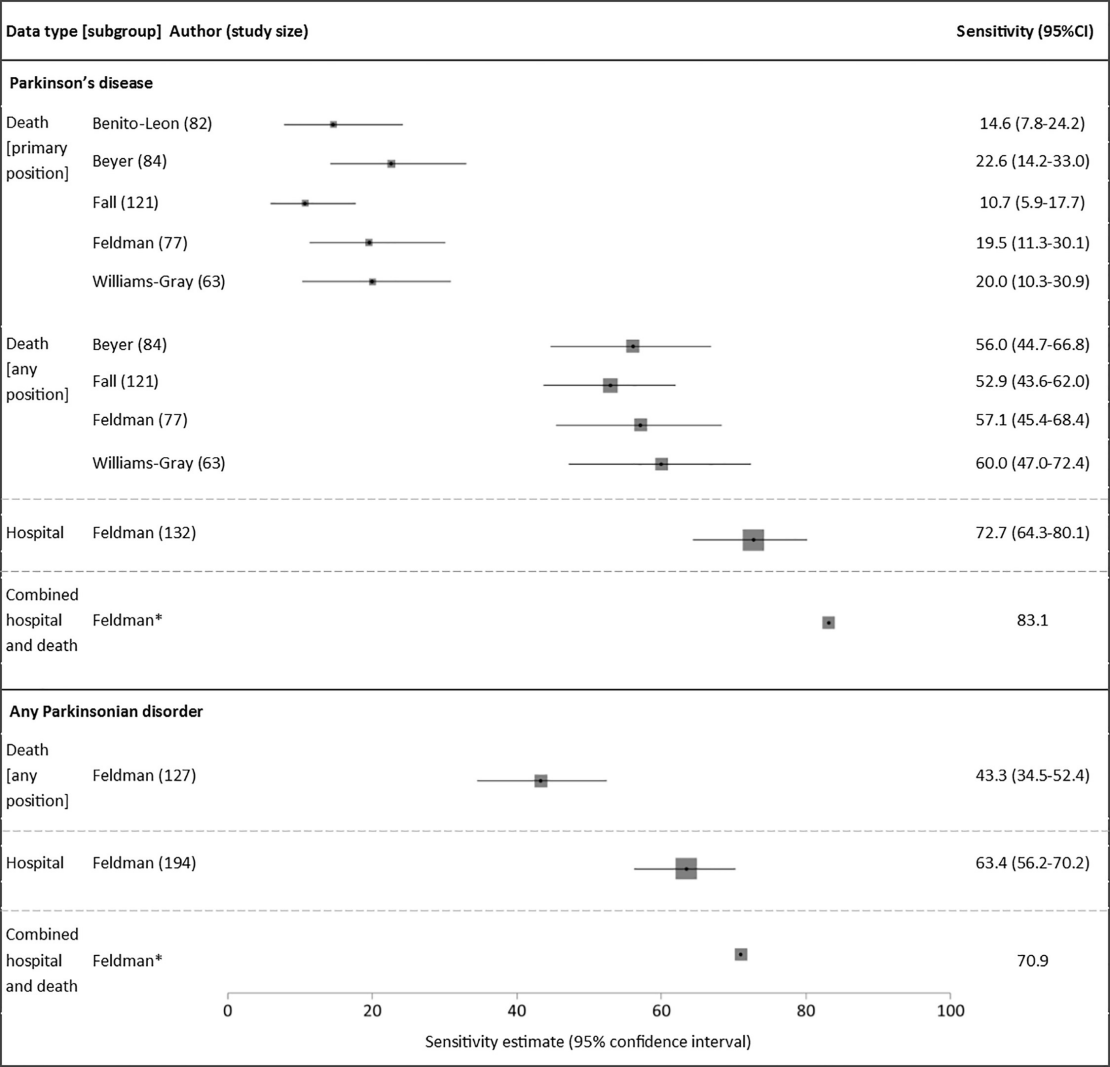
Sensitivity estimates of coded diagnoses. Study size: total number of true positives according to reference standard (true positives + false negatives). *Unknown sample size and confidence intervals. Box sizes reflect Mantel-Haenszel weight of study (inverse variance, fixed effects).

For PD, there were 11 sensitivity estimates in total (Fig 3)[24,37–40]. Of these, nine were sensitivity estimates for mortality data alone, consistently showing that codes in the primary position only gave low sensitivities of 11–23%, rising to 53–60% when codes from any position were included[24,37–40]. A single study reported the sensitivity of hospital data to be 73%, increasing to 83% when hospital and mortality data were combined. There were no sensitivity estimates for primary care or prescription data.

Of the two studies with low risk of bias, one investigated the sensitivity of mortality data, reporting a value of 20%. This was similar to the values reported by other studies deemed at risk of bias, suggesting that the potential bias identified did not significantly affect these estimates.

For parkinsonism, there were three sensitivity estimates, all from one study[24]. Hospital admissions and mortality data combined gave higher sensitivity (71%) compared with either mortality or hospital data alone (43% and 63% respectively).

## Discussion

We have demonstrated that existing validation studies show a wide variation in the accuracy of routinely collected healthcare data for the identification of PD and parkinsonism cases. Despite this, in some circumstances, achieving high PPVs is possible. Sensitivity (range 15–73% for PD) is generally lower than PPV (range 36–90%) in single datasets, but is increased by combining data sources.

When using routinely collected datasets to identify disease cases, there will inevitably be a trade-off between PPV and sensitivity[16]. The extent to which cohorts seek to maximise one accuracy metric over another will depend on the specific study setting and research question. For example, for studies that rely only on routinely collected data to identify disease cases are likely to desire a high PPV, providing sensitivity is sufficient to ensure statistical power in analyses. In contrast, for studies that use routinely collected data to identify potential cases before going onto validate these cases with a more detailed in-person or medical record review, a high sensitivity will be important. In this review, we found that the sensitivity of mortality data to detect PD using codes in the primary position alone was very low (range 11–23%) however, this markedly improved (range 56–60%) when codes were selected from any position on the death certificate[24,37–40]. No studies in this review investigated the effect of coding position on PPV, but previous studies of dementia and motor neurone disease have shown that selecting cases for whom the disease code was in the primary position consistently led to increased PPVs compared to selecting disease codes from any position[41–44]. However, as with PD, this approach led to the identification of fewer cases, thereby reducing sensitivity[17,18].

The pharmacological treatment of PD is largely focussed on improving motor function and patients are treated with a limited number of drugs. This has allowed antiparkinsonian drugs to be used as ‘tracers’ in epidemiological studies[45,46]. There are potential problems with using prescription data as a proxy for PD diagnosis. This approach may disproportionately under-identify patients with early stage disease who do not yet require treatment. Also, a response to a trial of dopaminergic drugs may be used as part of the diagnostic assessment in potential PD cases, meaning some patients prescribed antiparkinsonian medications will not be subsequently diagnosed with PD. Furthermore, antiparkinsonian can be prescribed for indications other than PD (such as dopamine agonists for restless legs syndrome, endocrine disorders and other forms of parkinsonism). The specific drugs licensed for use in parkinsonian conditions varies between countries and may change over time. Therefore, an algorithm incorporating prescription data would need to be continually revised to match prescribing patterns. Results from our review suggest that prescription data alone has a low PPV for PD case ascertainment[33]; however, when drug codes are combined with diagnostic codes, PPV increases but with reduced case ascertainment[28,32]. Furthermore, prescription datasets appear to have a higher PPV when identifying any parkinsonian disorder rather than specifically PD[33].

This study has several strengths and limitations. Our review benefits from prospective protocol publication, comprehensive search criteria, and independent duplication of each stage by two authors. Despite this, relevant studies may still have been missed, especially if a validation study was a subsection of a paper with a wider aim. As all eligible studies were included, the results may have been influenced by studies of lower quality. Only two articles were found to be at low risk of bias or applicability concerns[23,24], and it is likely that biases in study design would have affected the results. For example, one study with the lowest PPV[35] used very broad ICD-9 codes such as 781.0 (abnormal involuntary movements) and 781.3 (lack of coordination).

Since there is no method of diagnosing PD with certainty in life, there is likely to be some misclassification of the reference standards used in the studies. The application of stringent diagnostic criteria to reference standard diagnoses, although often necessary for research purposes, may lead to some patients being misclassified as ‘false positives’ when they do in fact have the condition. This may lead to underestimation of the PPV in some of the studies. When considering the ideal reference standard for validation studies, there is a trade-off between the robustness of the reference standard and validating sufficient cases to produce precise accuracy estimates. For example, in-person neurological examination may have greater diagnostic certainty than medical record review but this becomes difficult as the cohort size increases. Some of the variation in the reported results, therefore, is likely to be due to differences in how stringently different studies applied their reference standards.

Many of the studies reported cases with insufficient information to meet the reference standard and the handling of these varied. Some studies excluded such cases, others classified them as false positives, while some did not specify how they handled such missing data. Excluding such cases may introduce selection bias, whereas counting them as false positives may underestimate PPV.

The effect of possible publication bias on the results is difficult to estimate, but disproportionate publication of studies which report more favourable accuracy measures may lead to over-estimation of the performance of the codes. In addition, estimates of PPV are dependent upon the prevalence of the condition in the study population but it was not possible to assess the prevalence of PD within each study population.

Our review highlights several areas requiring further research. Given that the management of PD is largely delivered in outpatients or the community, primary care data may be an effective method of identifying cases. Whilst studies have suggested that PD diagnoses made in primary care are less accurate than those made in a specialist setting[47,48], primary care records combine notes made by primary care clinicians with prescription records and correspondence from secondary care. Codes from primary care should therefore include diagnoses made by specialists, thus increasing their accuracy. We found only one small study of primary care data, reporting a promising PPV of 81%, improving to 90% with the inclusion of medication codes[32]. No studies investigated the sensitivity of primary care data. Further research into the accuracy of primary care data is needed.

Two studies investigated using algorithmic combinations of codes from different sources to improve PPV[24,28]. These investigated the additional benefit of the inclusion of factors such as only including codes that appeared more than once, selecting codes in the primary position only, combining diagnostic codes with prescription data, and only including diagnoses made in specialist clinics. These methods increased PPV but at a cost to the number of cases identified. The development of algorithms that maximize PPV whilst maintaining a reasonable sensitivity (e.g., by combining multiple complimentary datasets) merits further evaluation.

To our knowledge, no studies have evaluated the accuracy of routinely collected healthcare data for solely identifying atypical parkinsonian syndromes such as PSP and MSA. Further work is needed to understand whether these datasets provide a valuable resource for studying these less common diseases.

In conclusion, our review summarises existing knowledge of the accuracy of routinely collected healthcare data for identifying PD and parkinsonism, and highlights approaches to increase accuracy and areas where further research is required. Given the wide range of observed results, prospective cohorts should perform their own validation studies where evidence is lacking for their specific setting.

## Supporting information

S1 Checklist PRISMA checklist. (DOC)

S1 File Search strategy. (DOCX)

S2 File QUADAS-2 assessment. (DOCX)

S1 Table QUADAS-2 summary results. (DOCX)
