# Supplementary material for: Identifying Parkinson's disease and parkinsonism cases using routinely collected healthcare data: A systematic review
Source: PLoS One. 2019 Jan 31;14(1):e0198736. doi: 10.1371/journal.pone.0198736 (PMC6354966; doi:10.1371/journal.pone.0198736)
Supplement: S1 File — (DOCX) [file pone.0198736.s002.docx]

**S1 File. Search strategy**

**MEDLINE search strategy (Ovid MEDLINE(R) In-Process & Other Non-Indexed Citations and Ovid MEDLINE(R) 1946 to Present)**

| 1 | "International Classification of Diseases"/ or "international classification of diseas*".mp. or "ICD ten*".mp. or ICD10.mp. or "ICD 10".mp. or "ICD 9".mp. or ICD9.mp. or "ICD nine".mp. or ICD-9-CM.mp. or ICD-10-CM.mp. or "administrat* data".mp. or "medical record*".mp. or "health information*".mp. or claim*.mp. or "hospital discharge*".mp. or "inpatient discharge*".mp. or "hospital episode*".mp. or "hospital episode statistics".mp. or "scottish morbidity record*".mp. or SMR*.mp. or "patient episode database for wales".mp. or PEDW.mp. or coding.mp. or code*.mp. or exp Clinical Coding/ or "medical record review".mp. or exp Information Systems/ or exp Medical Records/ or exp medical records systems, computerized/ or exp electronic health records/ or exp Electronic Health Records/ or exp Primary Health Care/ or exp general practice/ or exp family practice/ or "read cod*".mp. or exp Patient Discharge/ or exp Patient Discharge Summaries/ or exp Hospital Records/ or exp Health Services Research/ or "physician claims".mp. or "death certificate*".mp. or exp death certificates/ or exp hospital records/ or "death registration*".mp. or medicare.mp. or exp health insurance/ or exp Outpatients/ or "diagnostic and statistical manual".mp. or dsm*.mp. or "case ascertainment".mp. |
| --- | --- |
| 2 | (sensitivity or specificity).mp. or exp "sensitivity and specificity"/ or ((pre-test or pretest) adj probability).mp. or exp "Predictive Value of Tests"/ or "predictive value*".mp. or "likelihood ratio*".mp. or exp validation studies/ or "validation stud*".mp. or "positive predictive value".mp. or exp "reproducibility of results"/ or "reproducibility of results".mp. or "positive predictive value".mp. or "negative predictive value".mp. or validity.mp. or reproducibility.mp. or accuracy.mp. or agreement.mp. or validation.mp. or algorithm*.mp. or exp algorithms/ or (identif* adj3 dement*).ti,ab. or (detect* adj3 dement*).ti,ab. or (ROC or "receiver operat*").ab. or sROC.ab. or Area Under Curve/ |
| 3 | "Parkinson disease".mp. or exp Parkinson Disease/ or Parkinson*.mp. or "progressive supranuclear palsy".mp. or exp Supranuclear Palsy, Progressive/ or "Corticobasal Degeneration".mp. or Multiple System Atrophy.mp. or "vascular Parkinson*".mp. |
| 4 | 1 and 2 and 3 |
| 5 | limit 4 to yr="1990 –Current" |

**EMBASE search strategy – (Embase 1980 to Present)**

| 1 | "International Classification of Diseases"/ or disease classification/ or icd-10/ or icd-10-cm/ or icd-10-pcs/ or icd-9/ or icd-9-cm/ or "administrat* data".mp. or "medical record*".mp. or medical information system/ or medical informatics/ or electronic medical record/ or "health information".mp. or medical record/ or "hospital discharge*".mp. or claim*.mp. or "inpatient discharge*".mp. or "hospital episode*".mp. or "hospital episode statistics".mp. or "scottish morbidity record*".mp. or SMR.mp. or SMR01.mp. or "patient episode database for wales".mp. or PEDW.mp. or coding algorithm/ or patient coding/ or coding/ or coding.mp. or code*.mp. or "medical record review".mp. or exp medical record/ or exp electronic medical record/ or exp primary health care/ or "read cod*".mp. or "hospital record*".mp. or exp death certificate/ or registration/ or medicare.mp. or exp health insurance/ or exp Outpatients/ or "outpatient data".mp. or dsm*.mp. |
| --- | --- |
| 2 | "sensitivity and specificity"/ or exp predictive value/ or exp accuracy/ or validity/ or predictive validity/ or validation.mp. |
| 3 | exp Parkinsons/ or Parkinsons disease/ or vascular Parkinsons/ or multiple system atrophy/ or progressive supranuclear palsy/ or corticobasal degeneration |
| 4 | 1 and 2 and 3 |
| 5 | limit 4 to yr="1990 -Current" |

**Web of Science search strategy – (Web of Science Core Collection)**

| 1 | TS=("international classification of diseases" OR "ICD*" OR "administrat* data" OR "medical record*" OR "health information" OR claim* OR "hospital discharge*" OR "inpatient discharge*" OR "hospital episode*" OR "hospital episode statistics" OR "Scottish morbidity record" OR "patient episode database for wales" OR coding OR code* OR "medical record*" OR "electronic health record*" OR "hospital record*" OR medicare OR "health insurance") |
| --- | --- |
| 2 | TS=("positive predictive value" OR "negative predictive value" OR accuracy OR sensitivity OR specificity OR validity) |
| 3 | TS="Parkinson’s disease" OR "Parkinson’s" OR "Parkinson*" |
| 4 | 1 and 2 and 3 (*Timespan=1990-2017)* |

**Cochrane Library search strategy – (Cochrane Reviews (Reviews only), Other Reviews, Trials, Methods Studies, Technology Assessments, Economic Evaluations and Cochrane Groups)**

| 1 | "international classification of diseases" OR "ICD*" OR "administrat* data" OR "medical record*" OR "health information" OR claim* OR "hospital discharge*" OR "inpatient discharge*" OR "hospital episode*" OR "hospital episode statistics" OR "Scottish morbidity record" OR "patient episode database for wales" OR coding OR code* OR "medical record*" OR "electronic health record*" OR "hospital record*" OR medicare OR "health insurance" |
| --- | --- |
| 2 | "positive predictive value" OR "negative predictive value" OR accuracy OR sensitivity OR specificity OR validity |
| 3 | "Parkinson’s disease" OR Parkinson* OR MSA OR PSP OR CBD OR "vascular parkinson*" |
| 4 | 1 and 2 and 3 (Year from 1990 to 2017) |
