# Supplementary material for: Identifying Parkinson's disease and parkinsonism cases using routinely collected healthcare data: A systematic review
Source: PLoS One. 2019 Jan 31;14(1):e0198736. doi: 10.1371/journal.pone.0198736 (PMC6354966; doi:10.1371/journal.pone.0198736)
Supplement: S1 Table — (DOCX) [file pone.0198736.s004.docx]

**S3 Table - QUADAS-2 summary results**

| Risk of Bias | | | | | | | | | | | | Applicability Concerns | | | | | | |
| --- | --- | --- | --- | --- | --- | --- | --- | --- | --- | --- | --- | --- | --- | --- | --- | --- | --- | --- |
| First Author | | Patient Selection | | Index Test | | Reference Standard | | Flow and Timing | | Concern? | | Patient Selection | | Index Test | | Reference Standard | | Concern? |
| Benito-Leόn | | *√* | | ? | | *√* | | x | | Yes | | *√* | | ? | | *√* | | Yes |
| Beyer | | *√* | | ? | | ? | | *√* | | Yes | | *√* | | ? | | *√* | | Yes |
| Bower | | *√* | | x | | *√* | | *√* | | Yes | | *√* | | x | | *√* | | Yes |
| Butt | | *√* | | *√* | | *√* | | *√* | |  | | *√* | | *√* | | *√* | |  |
| Fall | | *√* | | ? | | *√* | | *√* | | Yes | | *√* | | ? | | *√* | | Yes |
| Feldman | | *√* | | *√* | | *√* | | *√* | |  | | *√* | | *√* | | *√* | |  |
| Gallo | | x | | *√* | | *√* | | x | | Yes | | ? | | *√* | | *√* | | Yes |
| Hernán | | *√* | | ? | | *√* | | *√* | | Yes | | *√* | | ? | | *√* | | Yes |
| Kestenbaum | | x | | *√* | | *√* | | *√* | | Yes | | x | | *√* | | *√* | | Yes |
| Meara | | *√* | | ? | | *√* | | ? | | Yes | | *√* | | ? | | *√* | | Yes |
| Savica | | *√* | | x | | *√* | | *√* | | Yes | | *√* | | x | | *√* | | Yes |
| Swarztrauber | | x | | *√* | | x | | x | | Yes | | x | | *√* | | x | | Yes |
| Szumski | | x | | *√* | | *√* | | *√* | | Yes | | x | | *√* | | *√* | | Yes |
| Thacker | | ? | | ? | | ? | | x | | Yes | | ? | | ? | | ? | | Yes |
| Wei | | x | | x | | ? | | *√* | | Yes | | x | | x | | ? | | Yes |
| Wermuth | | x | | *√* | | *√* | | *√* | | Yes | | *√* | | *√* | | *√* | |  |
| White | | x | | *√* | | *√* | | *√* | | Yes | | x | | *√* | | *√* | | Yes |
| Williams-Gray | | *√* | | ? | | *√* | | *√* | | Yes | | *√* | | ? | | *√* | | Yes |
| *√* | Low risk | |  | |  | |  | |  | |  | |  | |  | |  |  |
| ? | Unclear risk | |  | |  | |  | |  | |  | |  | |  | |  |  |
| x | High risk | |  | |  | |  | |  | |  | |  | |  | |  |  |
